# A Rare Cause of Urachal Adenocarcinoma: Urachal Diverticle

**DOI:** 10.1155/2013/571395

**Published:** 2013-02-07

**Authors:** Tufan Çiçek, Umut Gönülalan, Gökçen Çoban, Hilal Erinanç, Murat Koşan

**Affiliations:** ^1^Department of Urology, Faculty of Medicine, Başkent University, Hocacihan Mahallesi Saray Caddesi No. 1, Selcuklu, 42080 Konya, Turkey; ^2^Department of Radiology, Faculty of Medicine, Başkent University, 42080 Konya, Turkey; ^3^Department of Pathology, Faculty of Medicine, Başkent University, 42080 Konya, Turkey

## Abstract

Urachus is the remnant of the embryologic allantois and the fetal bladder, extending form the bladder roof to the umbilicus. It degenerates in the prenatal period into a tissue band known as the median umbilical ligament. Incomplete degeneration may lead to urachal diverticle development. It is difficult to diagnose unless it is considered in differential diagnosis and imaging modalities are employed. This paper describes a patient treated with partial cystectomy for urachal diverticle, and the pathologic examination revealed urachal adenocarcinoma.

## 1. Introduction 

Urachus is an embryologic remnant extending between the roof of the bladder and the umbilicus. It is obliterated by fibrosis in later fetal life. Incomplete closure of the vesical end of the urachus results in diverticle formation. Urachal pathologies are usually asymptomatic, rendering diagnosis difficult. They are symptomatic when they are complicated due to infection. Stones inside the intraurachal diverticle may lead to adenocarcinoma by irritation and infection [1]. Urachal diverticle-related adenocancer may be missed by cystoscopic methods [2]. The diagnosis must be verified histopathologically using imaging modalities.

## 2. Case Report

**Figure 1:**
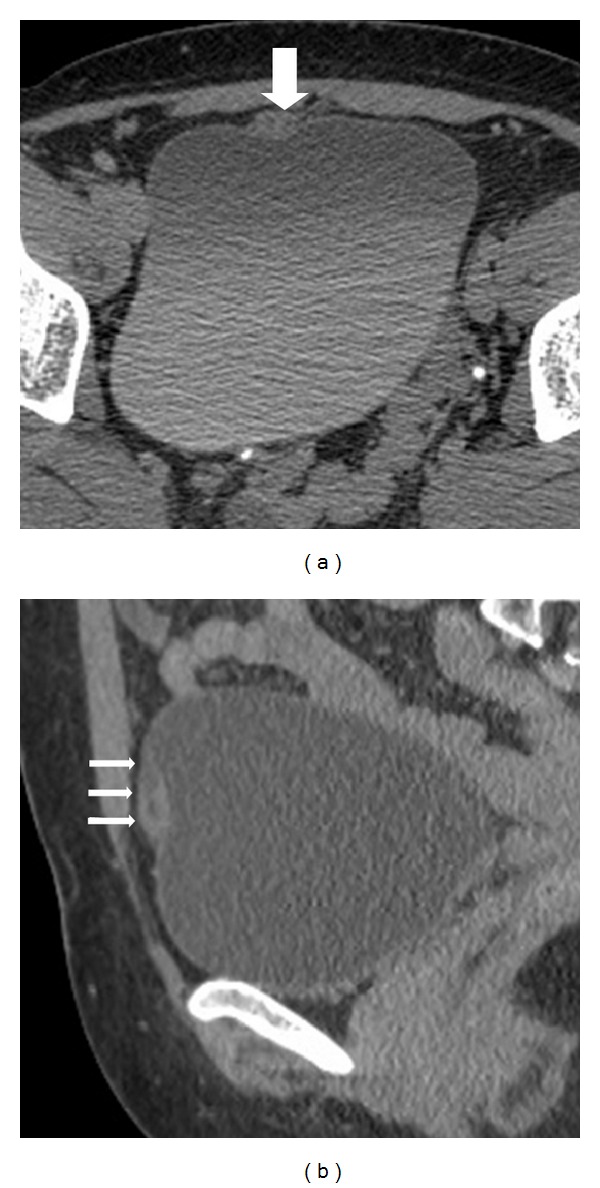
Contrast-enhanced CT revealed a fusiform mass with thick wall ((a), large white arrow on axial CT imaging) and internal millimetric cystic components overlying the anterosuperior portion of bladder ((b), small white arrows on sagittal CT imaging).

**Figure 2:**
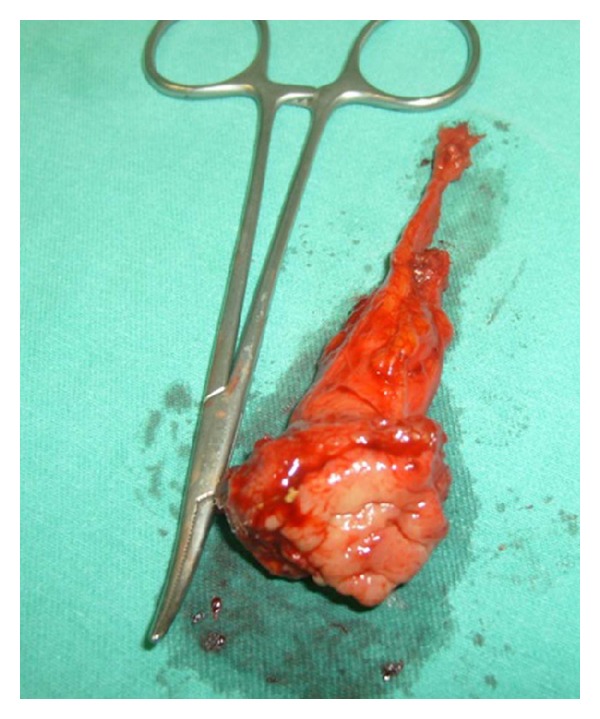
Partial cystectomy plus en bloc resection of the urachus.

**Figure 3:**
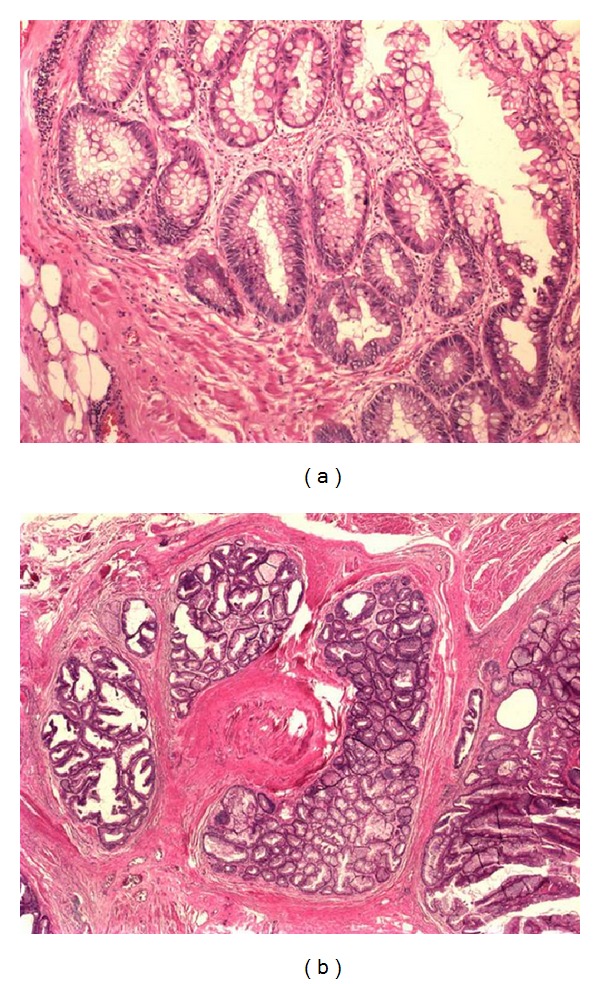
Microscopic examination revealed that the tumor has arised from a villous adenoma of the urachus. The pictures show that villous projections which were lined by columnar mucinous epithelium having pronounced nuclear and architectural atypia (a) and invasive area into the muscle wall (b).

A 43-year-old female with mucus excretion in urine for 4 years presented to our clinic following assessment in different centers. A former computerized tomography (CT) was consistent with a vesicourachal diverticle on the anterior wall of the urinary bladder (Figure 1). Physical examination was unremarkable except for a previous cesarean scar and suprapubic tenderness. A chest X-ray was normal. She was scheduled to undergo cystoscopy and appropriate intervention. Preoperative routines were sent. Urine examination showed 10 erythrocytes. Cystoscopy under the epidural anesthesia revealed no pathology on the roof of the bladder, except for an urachal ostium and mucus. A partial cystectomy operation was planned. By an incision from the previous Pfannenstiel incision, partial cystectomy, en bloc resection of the urachus, and excision of the parietal peritoneum were performed (Figure 2). After a 62-minute operation, the patient was hospitalized for 3 days. The urinary catheter was removed after 7 days. Histopathology of the partial cystectomy material revealed urachal adenocarcinoma with intact surgical borders (Figure 3). She was followed up with 3-month-interval cystoscopies. 

## 3. Discussion

Urachal pathologies may be classified into 4 main classes: patent urachus (50%), urachal cyst (30%), urachal sinus (15%), and urachal diverticle (5%) [2]. Infection is a common problem in these pathologies. Other complications include abscess formation, perforation, and tumor development [3].

This tumor is usually an adenocancer secreting mucin [4]. Bladder adenocancers may be of primary, urachal, and metastatic (ovarian, intestinal, prostate) origin. Urachal adenocarcinoma forms 0.5% of all adenocarcinomas [1].

The most common symptoms include hematuria [4]. In addition, nonspecific symptoms such as dysuria or hypogastric complaints may be present. The most common finding in patients undergoing endoscopy is an ulcerated or polypoid mass on the roof or anterior wall of the bladder [5]. In order to diagnose an adenocancer of urachus origin, the tumor should be on the roof or the anterior wall of the bladder and associated with the urachus; also there must be a sharp margin between the tumor and surface epithelium, and absence of cystitis glandularis and cystitis cystica [6]. Furthermore, metastatic tumors should be excluded. Since carcinomas are invasive against the muscular layer, TNM staging is not appropriate, and a specific staging system is employed [7] (Table 1). 

Radiologic modalities used in the diagnosis of urachal pathologies include radiography, cystogram, ultrasonography (USG), magnetic resonance imaging (MRI), and computerized tomography (CT) [4, 5]. Although abdominal radiography is frequently normal, it may rarely show psammomatous calcifications. Imaging methods like CT and MRI easily differentiate carcinomas located primarily on the roof of the bladder from urachal carcinomas growing outside the bladder [4]. 

Treatment of the urachal carcinomas is still debated. Surgical treatment remains the sole option for those who will not benefit radiotherapy and chemotherapy [8]. Surgical alternatives include partial or radical cystoprostatectomy, partial cystectomy plus umbilicotomy, and en bloc resection of the urachus. However, there is no significant difference between the organ-preserving surgeries and the radical surgery in terms of survival [9]. 

At the present time, both procedures may be performed via laparoscopic and robotic surgical methods. Laparoscopic partial cystectomy has the advantages of short hospital stay and fair cosmetic outcomes. However, long procedure times are still the main drawback [10].

The long-term outcomes with laparoscopic and robotic procedures will form the basis for future studies. However, since urachal adenocancers are considerably rare and minimal invasive treatment options are not available to large patient series, open surgical methods remain as the main treatment option. Urachal adenocancer should be absolutely remembered in patients presenting with mucinuria and diagnosed with urachal diverticle. No matter which treatment modality is employed, all methods should include a wide resection to prevent recurrences.

## Figures and Tables

**Table 1:** Clinical staging in urachal carcinomas.

Stage	Definition
I	Lesion limited to urachal mucosa
II	Lesion limited to urachus
III	Local extension
	(A) Local extension to bladder
	(B) Local extension to abdomen
	(C) Infiltration of the peritoneum
	(D) Extension to other local organs
IV	Metastatic extension
	(A) Regional lymph node
	(B) Distant metastasis
